# Supplementary material for: Potential pandemic risk of circulating swine H1N2 influenza viruses
Source: Nat Commun. 2024 Jun 13;15:5025. doi: 10.1038/s41467-024-49117-z (PMC11176300; doi:10.1038/s41467-024-49117-z)
Supplement: Supplementary file 1 — Supplementary information [file 41467_2024_49117_MOESM1_ESM.pdf]

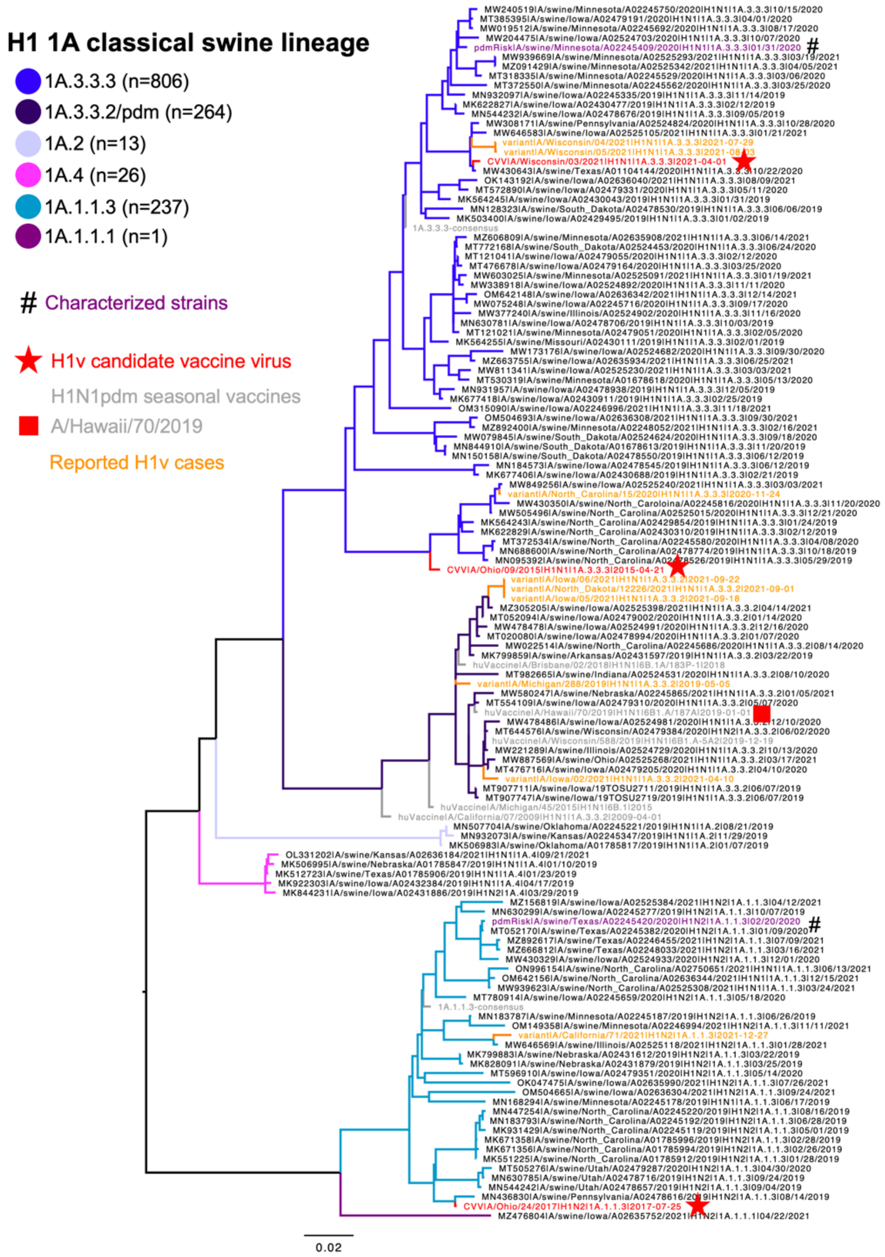

Supplementary Figure 1. Representative phylogenetic relationships of North American swine H1 1A classical swine lineage influenza A viruses from 2019 to 2021. Each genetic clade was proportionately down sampled using smot[64] and branches were colored. Swine influenza A virus strains characterized are marked by hash signs (#) and colored purple with the genetic clade consensus colored gray. The numbers in parentheses in the color key indicate number of each genetic clade detected between 2019 and 2021. Human seasonal H1 vaccine strains were colored gray; candidate vaccine viruses were colored red; and reported H1 variant cases detected between 2019 and 2021 were colored orange. The tree was midpoint rooted; all branch lengths are drawn to scale, and the scale bar indicates the number of nucleotide substitutions per site. The complete H1 phylogeny and input data are presented at https://github.com/flu-crew/datasets.

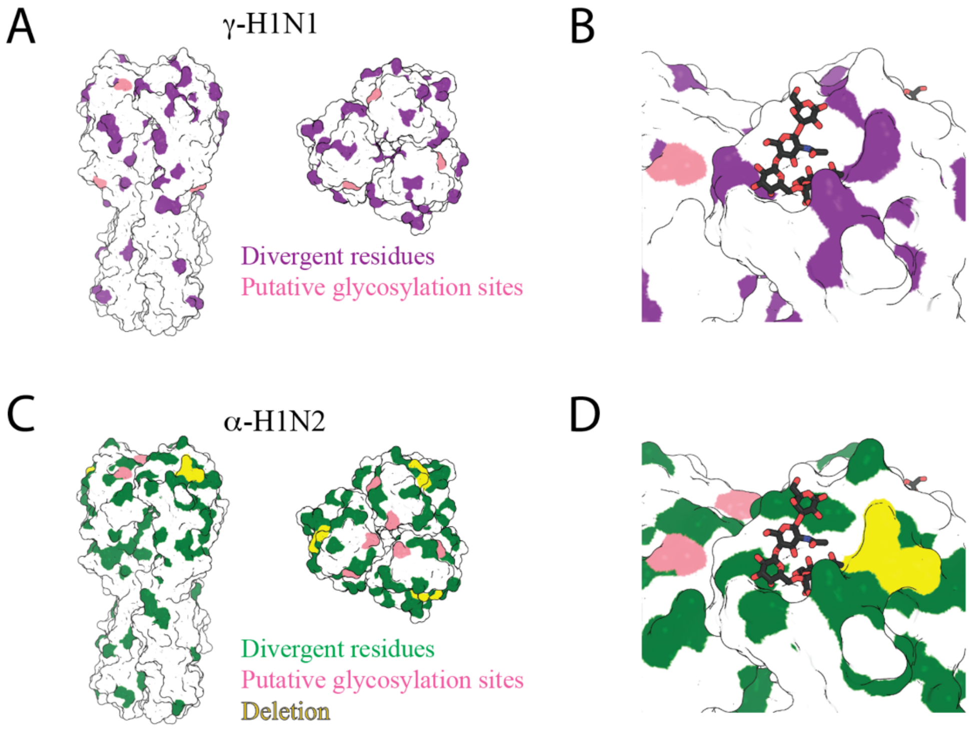

Supplementary Figure 2. HA structure comparison between H1N1pdm09 and swine viruses. (A) Top and side views of a surface representation of HA colored to compare the amino acid sequence of H1N1pdm09 HA and γ-swH1N1. Differences in amino acid sequences are represented in purple and differential putative glycosylation sites are colored pink. (B) γ-swH1N1 receptor binding site including α2-6-linked SA (sticks). (C) Top and side views of a surface representation of HA comparing the amino acid sequence of H1N1pdm09 HA and α-swH1N2. Differences in amino acid sequences are represented in green and differential putative glycosylation sites are colored pink. The two amino acid residue deletion at residue 133 and 133a (H3N2 numbering) in α-swH1N2 HA are highlighted in yellow. (D) α-swH1N2 receptor binding site including α2-6-linked SA (sticks). Images created in PyMOL and is based on PDB 3UBE.

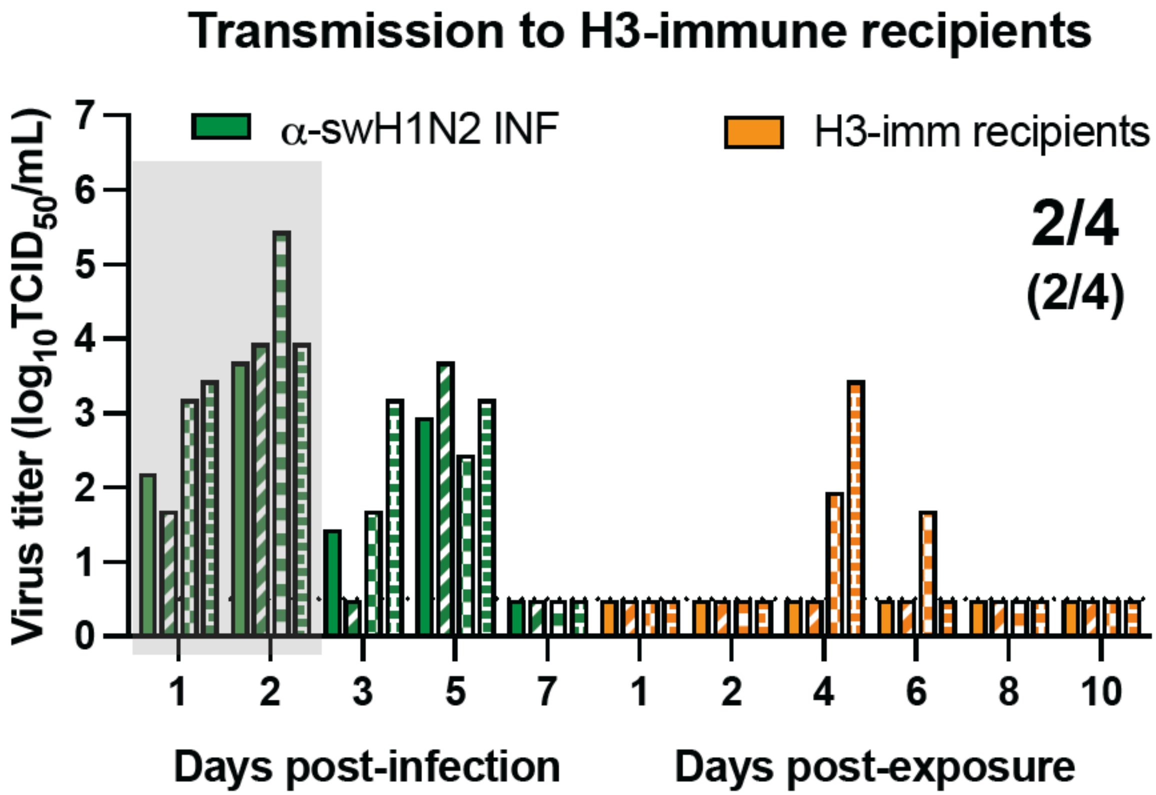

Supplementary Figure 3. α-swH1N2 transmission to H3N2-imm recipients. Four ferrets were infected with H3N2 A/Perth/16/2009 strain (H3N2-imm) 137 days prior to acting as recipients to α-swH1N2 infected donors. Four donor ferrets were infected with α-swH1N2 and H3N2-imm recipients were placed in the adjacent cage 24 hours later. Nasal washes were collected from all ferrets on the indicated days and titered for virus by $TCID_{50}$. Each bar indicates an individual ferret. For all graphs, the number of recipient ferrets with detectable virus in nasal secretions out of four total is shown; the number of recipient animals that seroconverted at 14- or 21-days post α-swH1N2 exposure out of four total is shown in parentheses. Gray shaded box indicates shedding of the donor during the exposure period. The limit of detection is indicated by the dashed line.

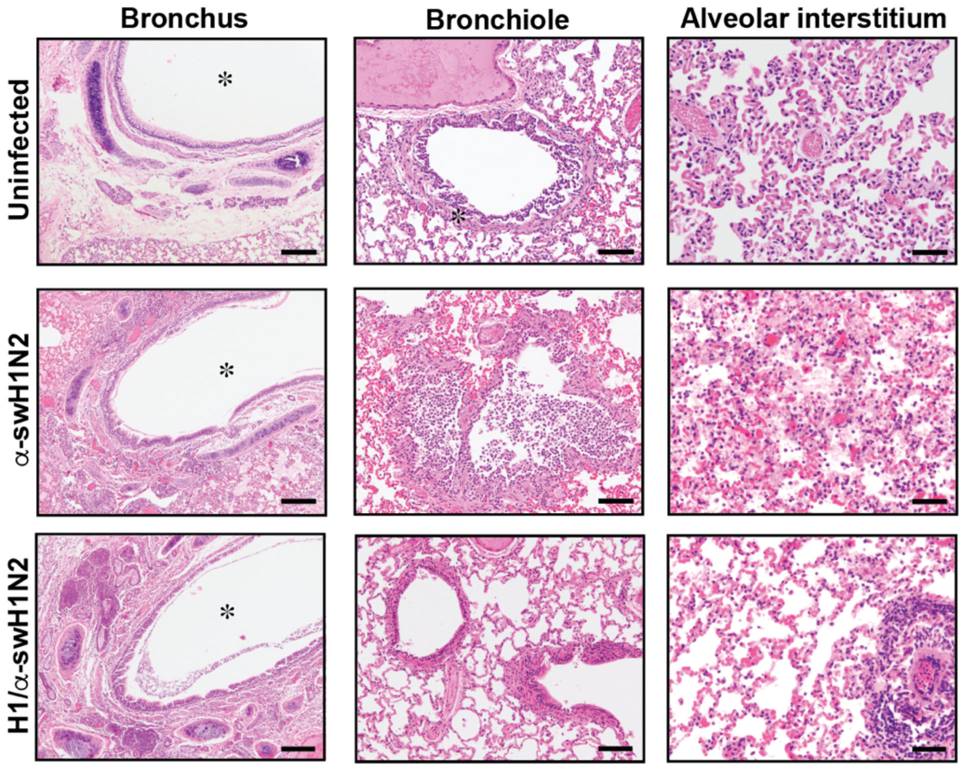

Supplementary Figure 4. Lung pathology caused by swine a-H1N2 influenza virus is more severe in ferrets with no prior immunity. Lungs from uninfected or α-swH1N2 infected ferrets without or with H1N1pdm09 pre-existing immunity (from Figure 7D) were harvested at 3 dpi. Histopathology was examined by H&E staining. N=2 for each group. Bronchus and bronchiole are at 20x magnification and alveolar interstitium is at 10x. Scale bar is 100 mm for bronchus and bronchiole and 200 mm for alveolar interstitium. Asterisks indicate the large airway.

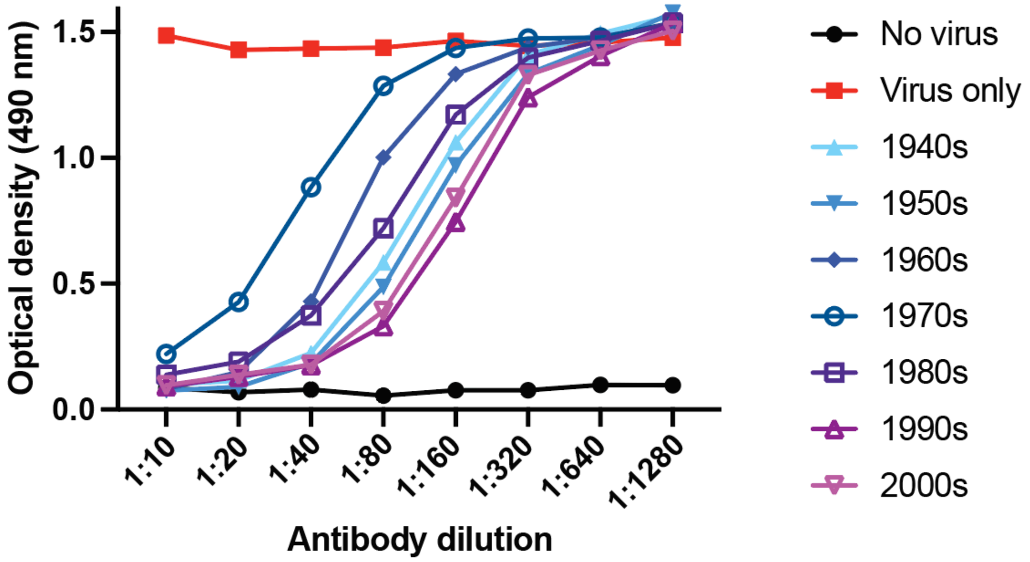

Supplementary Figure 5. Detection of human anti-NA antibodies that block NA activity. Serially diluted human sera, pooled by birth year, was incubated with an H9 reassortant bearing a NA antigens from A/swine/NY/A01104005/2011. Inhibition of NA activity was tested using fetuin substrate coated plates. Negative control with PBS only (no virus) and positive control with no serum were added as comparators. The data are representative of three experiments run in duplicate.

Supplementary Table 1: Glycan Structures on Microarray

	Catalogue	Structure	Symbol
1	**M040**	Galβ(1-4)-GlcNAcβ-ethyl-NH2	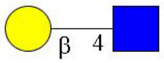
2	M221		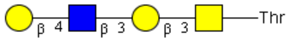
3	M222		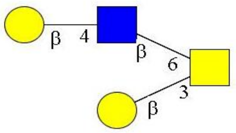
4	**M009**	Galβ(1-4)-GlcNAcβ(1-2)-Manα(1-3)-[Galβ(1-4)-GlcNAcβ(1-2)-Manα(1-6)]-Manβ(1-4)-GlcNAcβ(1-4)-GlcNAcβ-Asn-NH_2	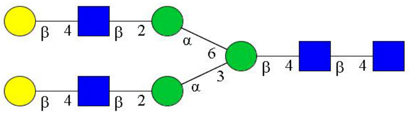
5	M226		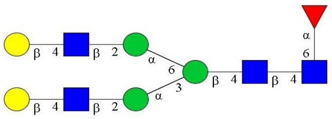
6	M227		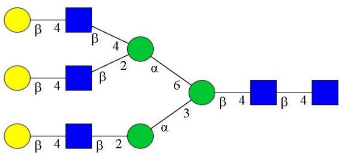
7	**SW29**	NeuAca(2-3)-Galβ(1-4)-GlcNAcβ-ethyl-NH_2	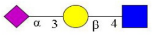

**8**	**SW30**	NeuAca(2-3)-Galβ(1-4)-GlcNAcβ(1-3)-Galβ(1-4)-GlcNAcβ-ethyl-NH_2	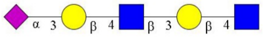
**9**	**SW31**	NeuAcα(2-3)-Galβ(1-4)-GlcNAcβ(1-3)-Galβ(1-4)-GlcNAcβ(1-3)-Galβ(1-4)-GlcNAcβ-ethyl-NH2	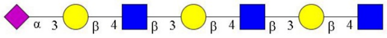
**10**	**M045**	NeuAcα(2-3)-Galβ(1-3)-GalNAcα-Thr-NH_2	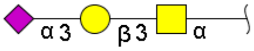
**11**	M120	3’ NeuAc LN Core 1 (1163)	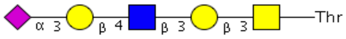
**12**	M128	3’ NeuAc DiLN Core 1 (1528)	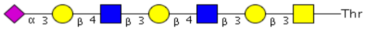
**13**	M142	3’ NeuAc TetraLN Core 1 (2259)	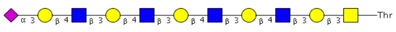
**14**	M143	3’ NeuAc PentaLN Core 1 (2624)	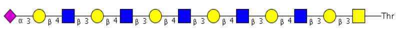
**15**	**M050**	NeuAcα(2-3)-Galβ(1-4)-GlcNAcβ(1-6)-[Galβ(1-3)]-GalNAcα-Thr-NH_2	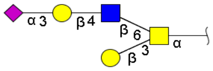

**16**	**M053**	NeuAcα(2-3)-Galβ(1-4)-GlcNAcβ(1-3)-Galβ(1-4)-GlcNAcβ(1-6)-[Galβ(1-3)]-GalNAcα-Thr-NH_2	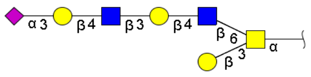
**17**	**M202**	3’ NeuAc TriLN Core 2 (1894)	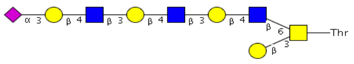
**18**	M152	3’ NeuAc TetraLN Core 2 (2259)	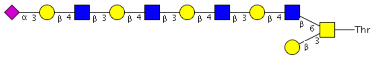
**19**	M149	3’ NeuAc PentaLN Core 2 (2624)	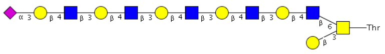
**20**	SW07	NeuAca2-3Galb1-4GlcNAcb1-2Mana1-3(NeuAca2-3Galb1-4GlcNAcb1-2Mana1-6)Manb1-4GlcNAcb1-4GlcNAc-AsnGly	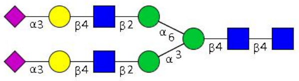
**21**	SW08	NeuAca2-3Galb1-4GlcNAcb1-3Galb1-4GlcNAcb1-2Mana1-3(NeuAca2-3Galb1-4GlcNAcb1-3Galb1-4GlcNAcb1-2Mana1-6)Manb1-4GlcNAcb1-4GlcNAc-AsnGly	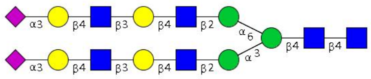
**22**	SW09	(NeuAca2-3Galb1-4GlcNAcb1-3Galb1-4GlcNAcb1-3Galb1-4GlcNAcb1-2Mana1-6)NeuAca2-3Galb1-4GlcNAcb1-3Galb1-4GlcNAcb1-3Galb1-4GlcNAcb1-2Mana1-3Manb1-4GlcNAcb1-4GlcNAc-AsnGly	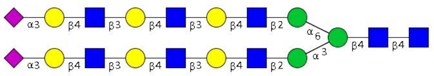
**23**	SW10	NeuAca2-3Galb1-4GlcNAcb1-2Mana1-3(NeuAca2-3Galb1-4GlcNAcb1-2Mana1-6)Manb1-4GlcNAcb1-4(Fuca1-6)GlcNAc-AsnGly	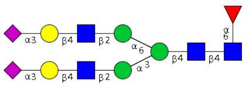
**24**	SW11	NeuAca2-3Galb1-4GlcNAcb1-3Galb1-4GlcNAcb1-2Mana1-3(NeuAca2-3Galb1-4GlcNAcb1-3Galb1-4GlcNAcb1-2Mana1-6)Manb1-4GlcNAcb1-4(Fuca1-6)GlcNAc-AsnGly	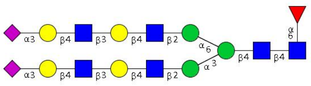
**25**	SW12	NeuAca2-3Galb1-4GlcNAcb1-3Galb1-4GlcNAcb1-3Galb1-4GlcNAcb1-2Mana1-3(NeuAca2-3Galb1-4GlcNAcb1-3Galb1-4GlcNAcb1-3Galb1-4GlcNAcb1-2Mana1-6)Manb1-4GlcNAcb1-4GlcNAc-AsnGly	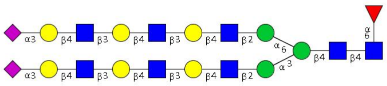

**26**	**M002**	NeuAca(2-3)-Galβ(1-4)-[Fuca(1-3)]-GlcNAcβ-propyl-NH_2	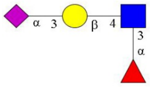
**27**	**M029**	NeuAca(2-3)-Galβ(1-3)-[Fuca(1-4)]-GlcNAcβ(1-3)-Galβ(1-4)-[Fuca(1-3)]-GlcNAcβ-ethyl-NH_2	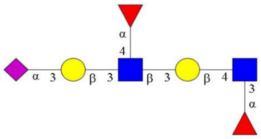
**28**	**M022**	NeuAca(2-3)-Galβ(1-4)-[Fuca(1-3)]-GlcNAcβ(1-3)-Galβ(1-4)-[Fuca(1-3)]-GlcNAcβ-ethyl-NH_2	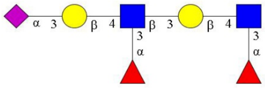
**29**	**M015**	NeuAca(2-3)-Galβ(1-4)-[Fuca(1-3)]-GlcNAcβ(1-3)-Galβ(1-4)-[Fuca(1-3)]-GlcNAcβ(1-3)-Galβ(1-4)-[Fuca(1-3)]-GlcNAcβ-ethyl-NH_2	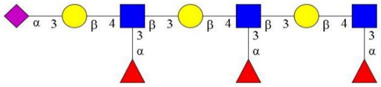
**30**	**M206**	3' SLeX TriLN Core 1(2332)	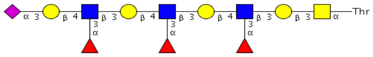
**31**	M147	3' SLeX TriLN Core 3(2170)	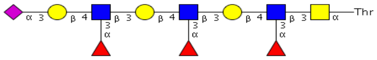
**32**	M215	NeuAc(2-6)-Galb(1-4)-(6S)GlcNacb-ethyl-NH2	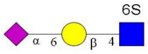
**33**	**M003**	NeuAca(2-6)-Galβ(1-4)-6-O-sulfo-GlcNAcβ-propyl-NH_2	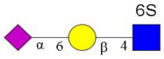

34	**SW32**	NeuAca(2-6)-Galβ(1-4)-GlcNAcβ-ethyl-NH_2	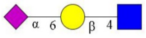
35	**SW33**	NeuAca(2-6)-Galβ(1-4)-GlcNAcβ(1-3)-Galβ(1-4)-GlcNAcβ-ethyl-NH_2	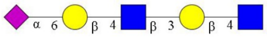
36	**SW34**	NeuAca(2-6)-Galβ(1-4)-GlcNAcβ(1-3)-Galβ(1-4)-GlcNAcβ(1-3)-Galβ(1-4)-GlcNAcβ-ethyl-NH_2	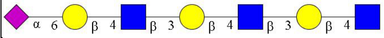
37	M121	6’ NeuAc LN Core 1 (1163)	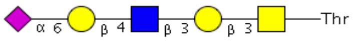
38	M129	6’ NeuAc DiLN Core 1 (1528)	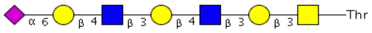
39	M154	6’ NeuAc TriLN Core 1 (1894)	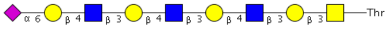
40	**M051**	NeuAcα(2-6)-Galβ(1-4)-GlcNAcβ(1-6)-[Galβ(1-3)]-GalNAcα-Thr-NH_2	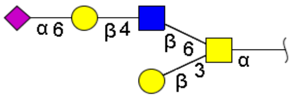
41	**M054**	NeuAcα(2-6)-Galβ(1-4)-GlcNAcβ(1-3)-Galβ(1-4)-GlcNAcβ(1-6)-[Galβ(1-3)]-GalNAcα-Thr-NH_2	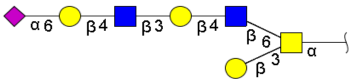

42	**M201**	6' NeuAc TriLN Core 2 (1894)	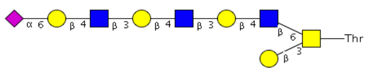
43	M159	6' NeuAc TetraLN Core 2 (2259)	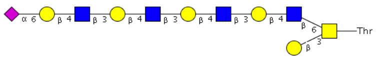
44	M157	6' NeuAc PentaLN Core 2 (2624)	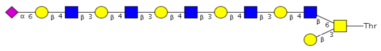
45	SW01	NeuAca2-6Galb1-4GlcNAcb1-2Mana1-3(NeuAca2-6Galb1-4GlcNAcb1-2Mana1-6)Manb1-4GlcNAcb1-4GlcNAc-AsnGly	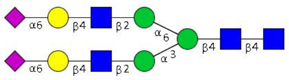
46	SW02	NeuAca2-6Galb1-4GlcNAcb1-3Galb1-4GlcNAcb1-2Mana1-3(NeuAca2-6Galb1-4GlcNAcb1-3Galb1-4GlcNAcb1-2Mana1-6)Manb1-4GlcNAcb1-4GlcNAc-AsnGly	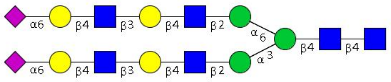
47	SW03	NeuAca2-6Galb1-4GlcNAcb1-3Galb1-4GlcNAcb1-3Galb1-4GlcNAcb1-2Mana1-3(NeuAca2-6Galb1-4GlcNAcb1-3Galb1-4GlcNAcb1-3Galb1-4GlcNAcb1-2Mana1-6)Manb1-4GlcNAcb1-4GlcNAc-AsnGly	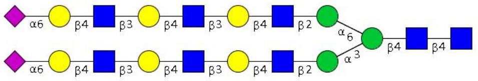
48	SW04	NeuAca2-6Galb1-4GlcNAcb1-2Mana1-3(NeuAca2-6Galb1-4GlcNAcb1-2Mana1-6)Manb1-4GlcNAcb1-4(Fuca1-6)GlcNAc-AsnGly	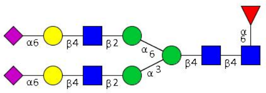
49	SW05	NeuAca2-6Galb1-4GlcNAcb1-3Galb1-4GlcNAcb1-2Mana1-3(NeuAca2-6Galb1-4GlcNAcb1-3Galb1-4GlcNAcb1-2Mana1-6)Manb1-4GlcNAcb1-4(Fuca1-6)GlcNAc-AsnGly	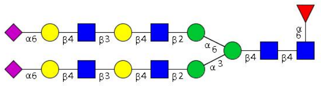
50	SW06	NeuAca2-6Galb1-4GlcNAcb1-3Galb1-4GlcNAcb1-3Galb1-4GlcNAcb1-2Mana1-3(NeuAca2-6Galb1-4GlcNAcb1-3Galb1-4GlcNAcb1-3Galb1-4GlcNAcb1-2Mana1-6)Manb1-4GlcNAcb1-4GlcNAc-AsnGly	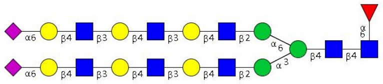

Supplementary Table 2: Serology of donor and recipient ferrets for each transmission study

Figure	Virus	Status	Transmission efficiency	α-swH1N2 MN titers (D0)	α-swH1N2 MN titers (D14)	α-swH1N2 MN titers (D21)	H1N1pdm09/H3N2 MN titers (D0)	H1N1pdm09/H3N2 MN titers (D14)	H1N1pdm09/H3N2 MN titers (D21)
5A	α-swH1N2	INF		<20, <20, <20, <20	80, 113, 320, 202	NA	ND	ND	ND
		Naïve	4/4	<20, <20, <20, <20	113, 40, 80, 80	1016, 806, 905, 905	NA	NA	NA
5B	α-swH1N2	INF		NA	NA	NA	NA	NA	NA
		H3N2-imm	4/4	<20, <20, <20, <20	403, 453, 453, 453	905, 806, 1280, 640	226, 254, 1016, 320	2032, 905, 1016, 806	3620, 806, 320, 640
5C	α-swH1N2	INF		NA	NA	NA	ND	ND	ND
		H1N1pdm09-imm	4/4	<20, <20, <20, <20	50, 40, 40, 20	2560, 3620, 640, 905	7241, 2560, 2560, 2032	12902, 14482, 10240, 14482	14482, 14482, 7241, 12902
6B	α-swH1N2	INF		NA	NA	ND	ND	ND	ND
		H1N1pdm09-imm	2/4	<20, <20, <20, <20	1016, 806, <20, <20	1613, 640, <20, <20	1280, 640, 3225, 3225	14482, 10240, 2560, 2560	8127, 10240, 2560, 3620
		Naïve	1/4	<20, <20, <20, <20	<20, 403, <20, <20	<20, 640, <20, <20	NA	NA	NA
6C	α-swH1N2	INF		<20, <20, <20, <20	3620, 3225, 1613, 2032	ND	ND	ND	ND
		H1N1pdm09-imm	4/4	<20, <20, <20, <20	508, 905, 806, 905	806, 1280, 905, 1810	1280, 1280, 1280, 5120	14482, 14482, 14482, 14482	12902, 7241, 10240, 14482
		Naïve	2/4	<20, <20, <20, <20	<20, <20, 1613, 806	<20, <20, 1280, 905	NA	NA	NA
6D	α-swH1N2	INF		<20, <20, <20, <20	905, 508, 640	ND	ND	ND	ND
		H1N1pdm09-imm	3/3	<20, <20, <20, <20	453, 905, 254	806, 1016, 403	1810, 1810, 3225	14482, 14482, 7241	10240, 14482, 6451
		H1N1pdm09-imm	1/3	<20, <20, <20, <20	<20, 403, <20	<20, 905, <20	2032, 2560, 3620	2560, 14482, 3620	2560, 14482, 3225
S3	α-swH1N2	INF		NA	NA	NA	ND	ND	ND
		H3N2-imm	2/4	<20, <20, <20, <20	<20, <20, 508, 1810	<20, <20, 1016, 403	453, 453, 3225, 403	508, 640, 14482, 806	508, 453, 14482, 2032

ND = Not determined; NA = Not available, as animals were euthanized at endpoint prior to day 14
MN = microneutralization
